# Supplementary material for: ProteoAutoNet: high-throughput co-eluted protein analysis with robotics and machine learning
Source: Nat Commun. 2026 Jan 22;17:1949. doi: 10.1038/s41467-026-68686-9 (PMC12929803; doi:10.1038/s41467-026-68686-9)

Supplementary Information

Supplementary Fig. 1 Performance evaluation of ProteoAutoNet for sample processing.

(A) The consistency of protein IDs was measured by Jaccard similarity index across three technical replicates. Range: 0-1, and 1 indicates perfect overlap. **(B)** The protein counts between manual and robotical processing groups in eight replicates of TPC-1 lysates, the number of proteins was annotated above the bars.

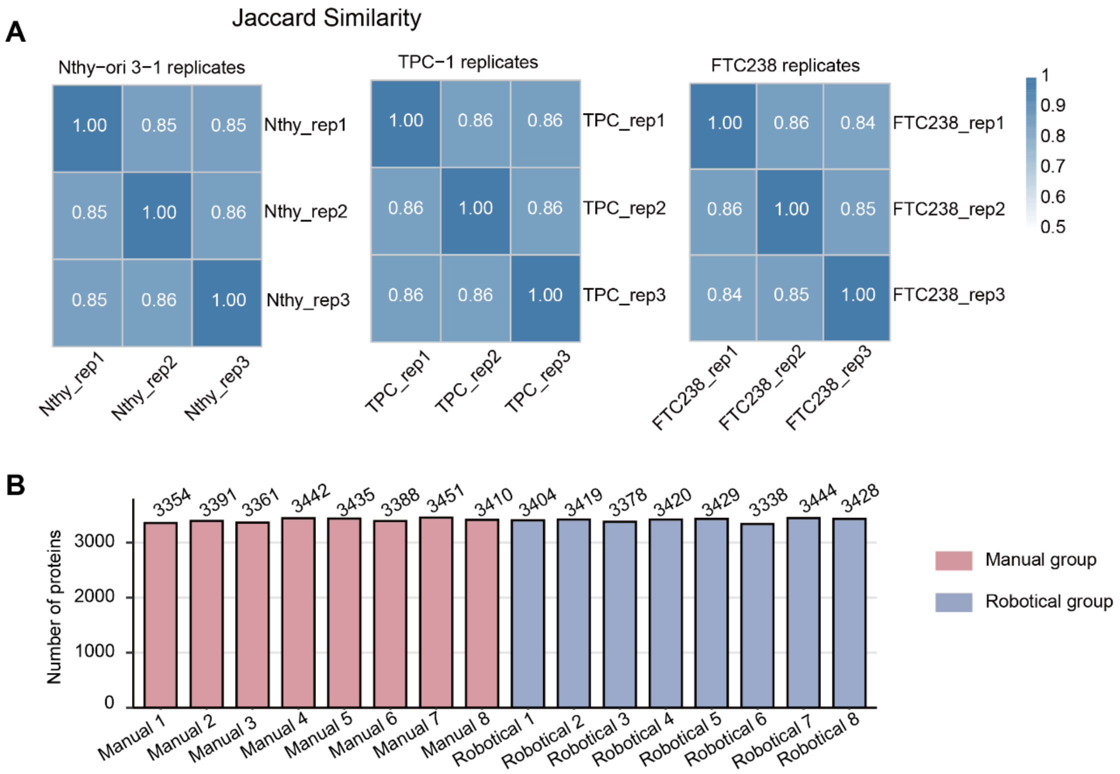

Supplementary Fig. 2 Data processing and benchmark evaluation for interaction prediction. (A) The number of identified proteins in each biological replicate of the Nthy-ori 3-1, TPC-1 and FTC238 cell lines. **(B)** The precision curve of the random forest model with a 1:5 negative-to-positive sampling ratio, showing its precision stability. The color of each fold is shown on the right, with the x-axis representing the number of interactions and the y-axis indicating the precision. **(C)** The distribution of scores under the 1:5 sampling ratio across three cell lines, showing the enrichment of high-confidence interactions (score > 0.5). **(D)** Representative protein trace showing data augmentation via value perturbation and missing value perturbation. Value perturbation was applied by scaling the original values to 90% (O43592 in Perturbation 1) and 110% (Q86Y56 in Perturbation 2). Missing values were imputed in both O43592 and Q86Y56. **(E)** Weighted precision of interactions derived from three cell lines. **(F)** Overlap of protein interactions across three cell lines. Predicted interactions are shown in black. Known interactions retrieved from the curated database are highlighted in red (in parentheses). **(G)** ROC curve for the external validation set from FTC133 cell line, yielding an AUC of 0.68.

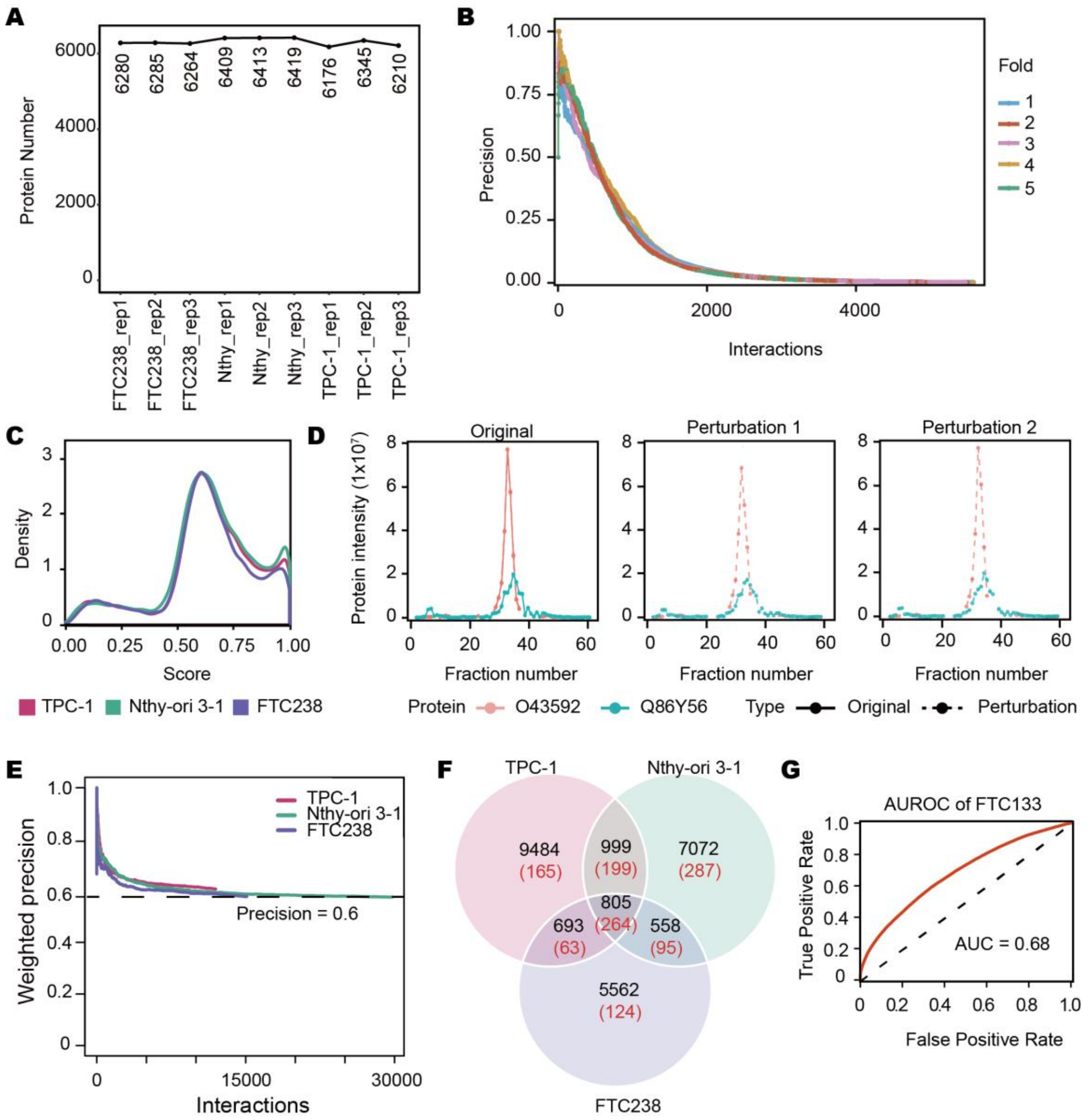

A
Protein Number
6280
6285
6264
6409
6413
6419
6176
6345
6210
FTC238_rep1
FTC238_rep2
FTC238_rep3
Nthy_rep1
Nthy_rep2
Nthy_rep3
TPC-1_rep1
TPC-1_rep2
TPC-1_rep3
B
Precision
Interactions
Fold
C
Density
Score
TPC-1
Nthy-ori 3-1
FTC238
D
Original
Perturbation 1
Perturbation 2
Protein intensity (1x10⁷)
Fraction number
Protein
O43592
Q86Y56
Type
Original
Perturbation
E
Weighted precision
Interactions
Precision = 0.6
F
TPC-1
Nthy-ori 3-1
FTC238
9484
(165)
999
(199)
7072
(287)
805
(264)
693
(63)
558
(95)
5562
(124)
G
AUROC of FTC133
True Positive Rate
False Positive Rate
AUC = 0.68

Supplementary Fig. 3 Protein interaction networks of key pathways from KEGG and GO databases. (A) Proteasome components identified across the three cell lines were mapped to the KEGG database, with 37 out of 43 proteins detected. The color of nodes indicates the cell line origin of each protein. The edges represent whether interactions were identified by CF-DIA-MS only or were supported by both CF-DIA-MS and the STRING database. Grey: detected by CF-DIA-MS. Red: CF-DIA-MS and included in STRING. **(B)** Sulfur metabolism pathway components were identified, with 7 out of 10 known proteins detected. Node and edge representations follow the same scheme as in panel A. **(C)** The protein interaction networks (PINs) of DNA replication (22 out of 36 known components). The PINs were clustered with the Markov clustering (MCL) algorithm. Node and edge representations follow the same scheme as in panel A. **(D, E)** The PINs of the ribosomal pathway (72 out of 131 known components). Clustering method and legend scheme are consistent with panel A. **(F)** The PINs of 2-oxocarboxylic acid metabolism (10 out of 18 known components). The clustering method and legend scheme are consistent with panel A. **(G)** The common PINs derived from three cell lines. The clustering method is the same as panel A. GO enrichment and protein interactions supported by both CF-DIA-MS and STRING databases are highlighted. Nodes: proteins expressed in all three cell lines. Dashed line: the interactions archived in STRING. Solid line: the interactions detected by CF-DIA-MS. Red double line: the interactions identified by CF-DIA-MS and corroborated by the STRING.

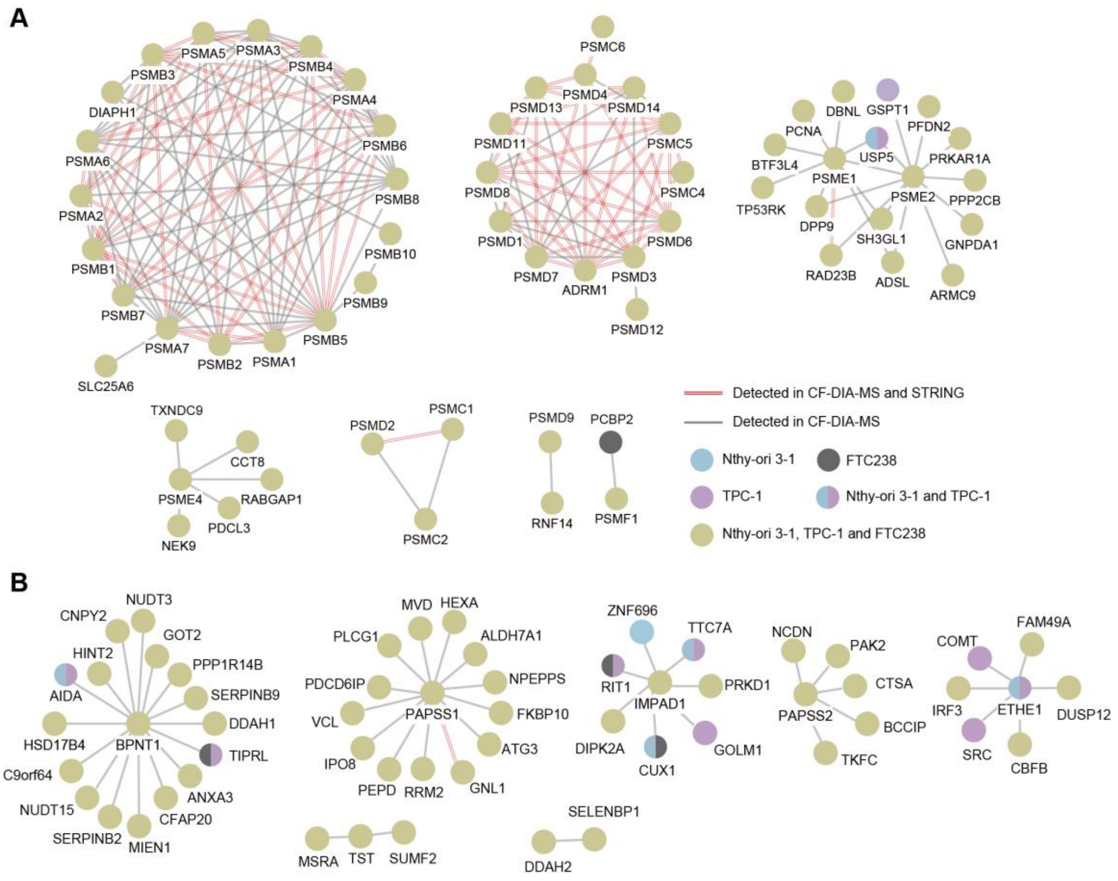

C

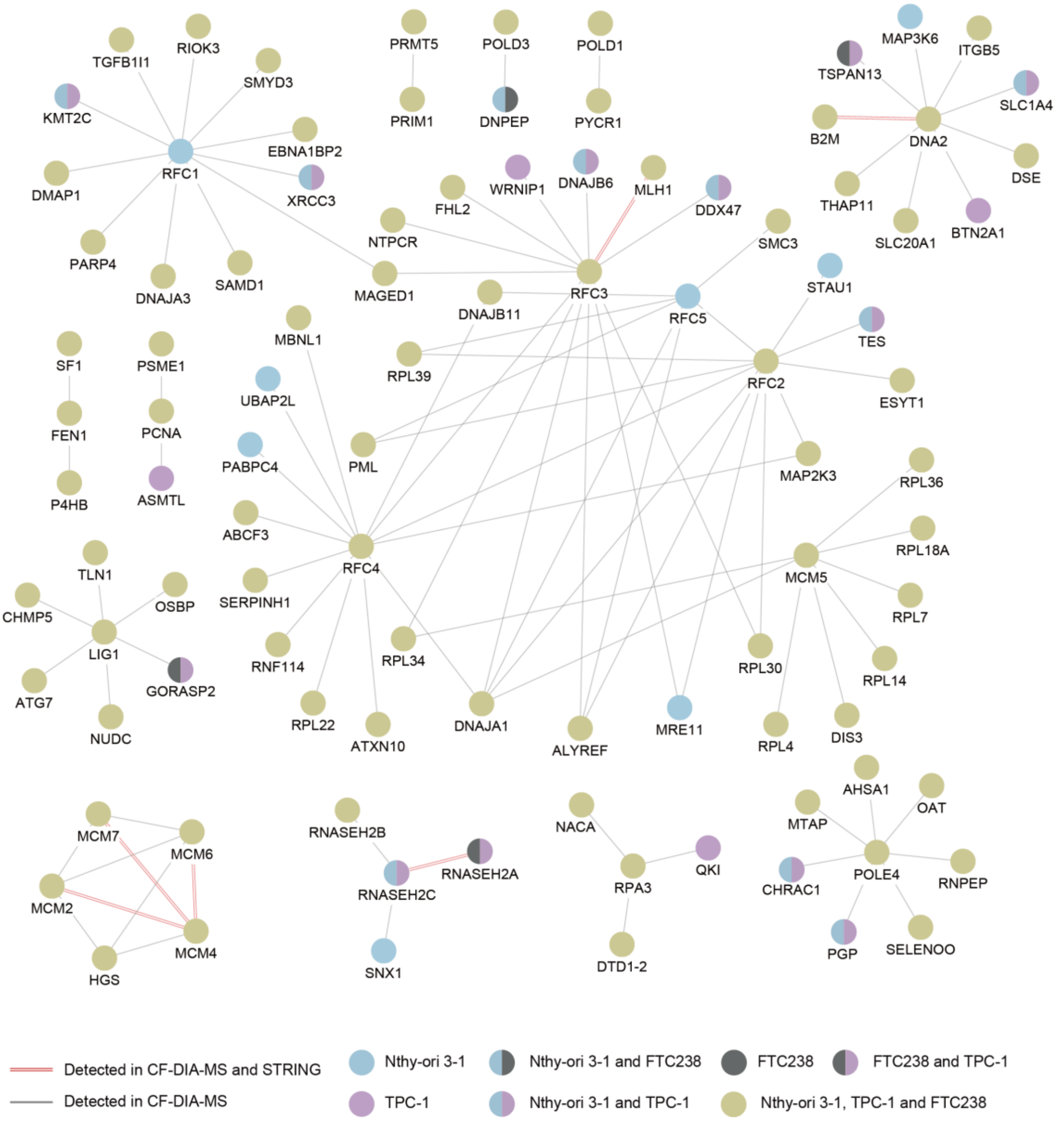
TGFB1I1
RIOK3
SMYD3
KMT2C
EBNA1BP2
RFC1
DMAP1
XRCC3
PARP4
DNAJA3
SAMD1
PRMT5
PRIM1
POLD3
DNPEP
POLD1
PYCR1
WRNIP1
DNAJB6
MLH1
FHL2
DDX47
NTPCR
MAGED1
RFC3
DNAJB11
RFC5
SMC3
MAP3K6
ITGB5
TSPAN13
SLC1A4
B2M
DNA2
DSE
THAP11
SLC20A1
BTN2A1
STAU1
TES
RFC2
ESYT1
SF1
FEN1
P4HB
PSME1
PCNA
ASMTL
MBNL1
RPL39
UBAP2L
PABPC4
PML
ABCF3
RFC4
SERPINH1
MAP2K3
RPL36
RPL18A
MCM5
RPL7
RPL30
RPL14
TLN1
CHMP5
OSBP
LIG1
GORASP2
ATG7
NUDC
RNF114
RPL34
RPL22
ATXN10
DNAJA1
ALYREF
MRE11
RPL4
DIS3
AHSA1
OAT
MTAP
CHRAC1
POLE4
RNPEP
PGP
SELENOO
MCM7
MCM6
MCM2
MCM4
HGS
RNASEH2B
RNASEH2A
RNASEH2C
SNX1
NACA
RPA3
QKI
DTD1-2
Detected in CF-DIA-MS and STRING
Detected in CF-DIA-MS
Nthy-ori 3-1
Nthy-ori 3-1 and FTC238
FTC238
FTC238 and TPC-1
TPC-1
Nthy-ori 3-1 and TPC-1
Nthy-ori 3-1, TPC-1 and FTC238

D

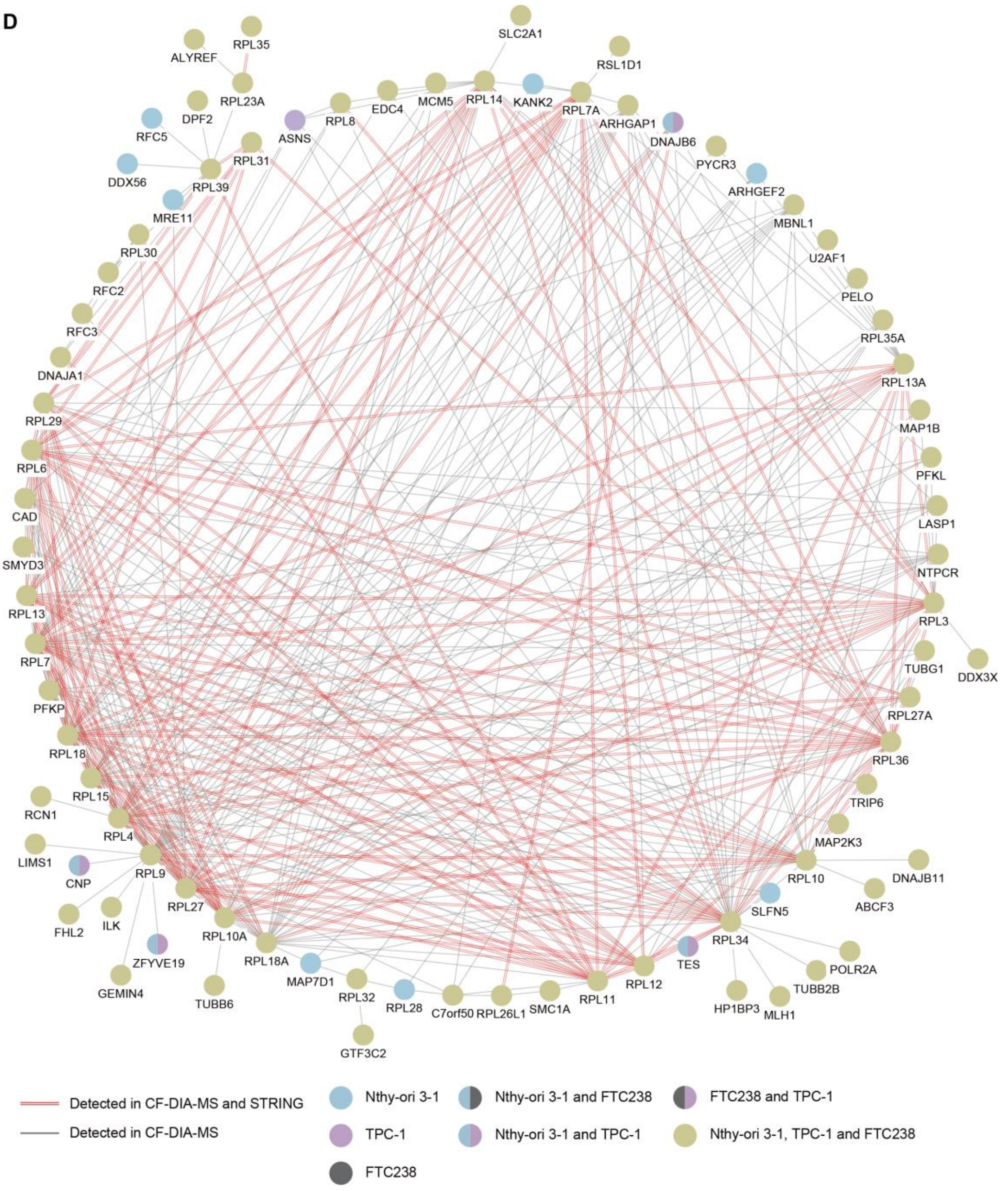

SLC2A1
RSL1D1
ALYREF
RPL35
RPL23A
DPF2
RFC5
ASNS
RPL8
EDC4
MCM5
RPL14
KANK2
RPL7A
ARHGAP1
DNAJB6
PYCR3
ARHGEF2
MBNL1
U2AF1
PELO
RPL35A
RPL13A
MAP1B
PFKL
LASP1
NTPCR
RPL3
DDX3X
TUBG1
RPL27A
RPL36
TRIP6
MAP2K3
RPL10
DNAJB11
ABCF3
SLFN5
RPL34
TES
POLR2A
TUBB2B
MLH1
HP1BP3
RPL12
RPL11
SMC1A
RPL26L1
C7orf50
RPL28
RPL32
GTF3C2
MAP7D1
RPL18A
TUBB6
RPL10A
RPL27
ZFYVE19
GEMIN4
ILK
FHL2
CNP
LIMS1
RPL9
RPL4
RCN1
RPL15
RPL18
PFKP
RPL7
RPL13
SMYD3
CAD
RPL6
RPL29
DNAJA1
RFC3
RFC2
RPL30
MRE11
RPL39
DDX56
RPL31
Detected in CF-DIA-MS and STRING
Detected in CF-DIA-MS
Nthy-ori 3-1
TPC-1
FTC238
Nthy-ori 3-1 and FTC238
Nthy-ori 3-1 and TPC-1
FTC238 and TPC-1
Nthy-ori 3-1, TPC-1 and FTC238

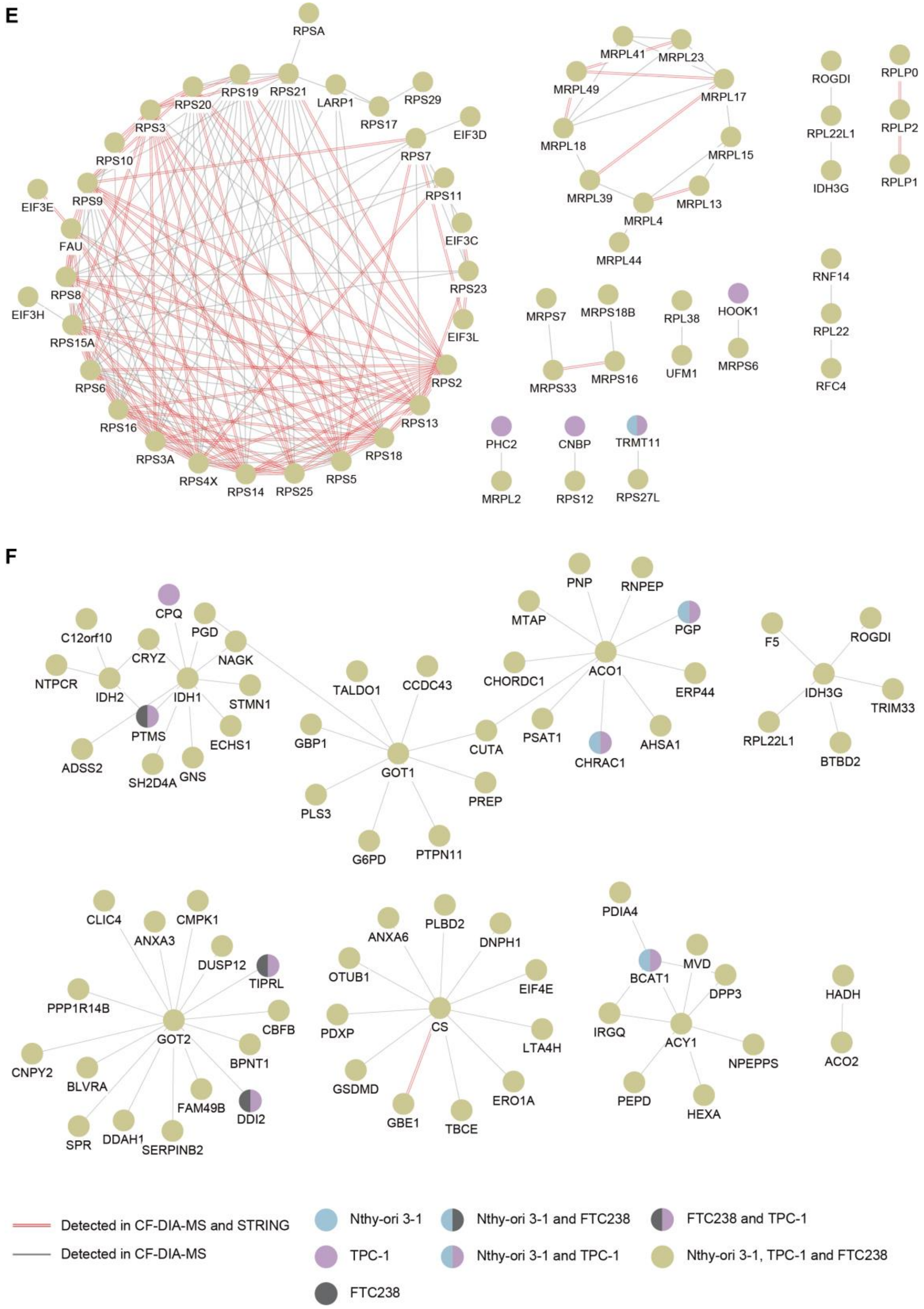

E
RPSA
RPS19
RPS21
RPS20
LARP1
RPS29
RPS3
RPS17
EIF3D
RPS10
RPS7
RPS11
EIF3E
RPS9
FAU
EIF3C
RPS23
RPS8
EIF3H
EIF3L
RPS15A
RPS2
RPS6
RPS13
RPS16
RPS18
RPS3A
RPS4X
RPS14
RPS25
RPS5
MRPL41
MRPL23
MRPL49
MRPL17
MRPL18
MRPL15
MRPL39
MRPL13
MRPL4
MRPL44
ROGDI
RPL22L1
IDH3G
RPLP0
RPLP2
RPLP1
RNF14
RPL22
RFC4
MRPS7
MRPS18B
MRPS33
MRPS16
RPL38
UFM1
HOOK1
MRPS6
PHC2
MRPL2
CNBP
RPS12
TRMT11
RPS27L
F
CPQ
PGD
C12orf10
CRYZ
NAGK
NTPCR
IDH2
IDH1
STMN1
PTMS
ECHS1
ADSS2
SH2D4A
GNS
TALDO1
CCDC43
GBP1
GOT1
CUTA
PREP
PLS3
G6PD
PTPN11
PNP
RNPEP
MTAP
PGP
CHORDC1
ACO1
ERP44
PSAT1
CHRAC1
AHSA1
F5
ROGDI
IDH3G
TRIM33
RPL22L1
BTBD2
CLIC4
CMPK1
ANXA3
DUSP12
TIPRL
PPP1R14B
GOT2
CBFB
BPNT1
CNPY2
BLVRA
FAM49B
DDI2
SPR
DDAH1
SERPINB2
ANXA6
PLBD2
DNPH1
OTUB1
EIF4E
PDXP
CS
LTA4H
GSDMD
ERO1A
GBE1
TBCE
PDIA4
BCAT1
MVD
DPP3
IRGQ
ACY1
NPEPPS
PEPD
HEXA
HADH
ACO2
Detected in CF-DIA-MS and STRING
Detected in CF-DIA-MS
Nthy-ori 3-1
TPC-1
FTC238
Nthy-ori 3-1 and FTC238
Nthy-ori 3-1 and TPC-1
FTC238 and TPC-1
Nthy-ori 3-1, TPC-1 and FTC238

G

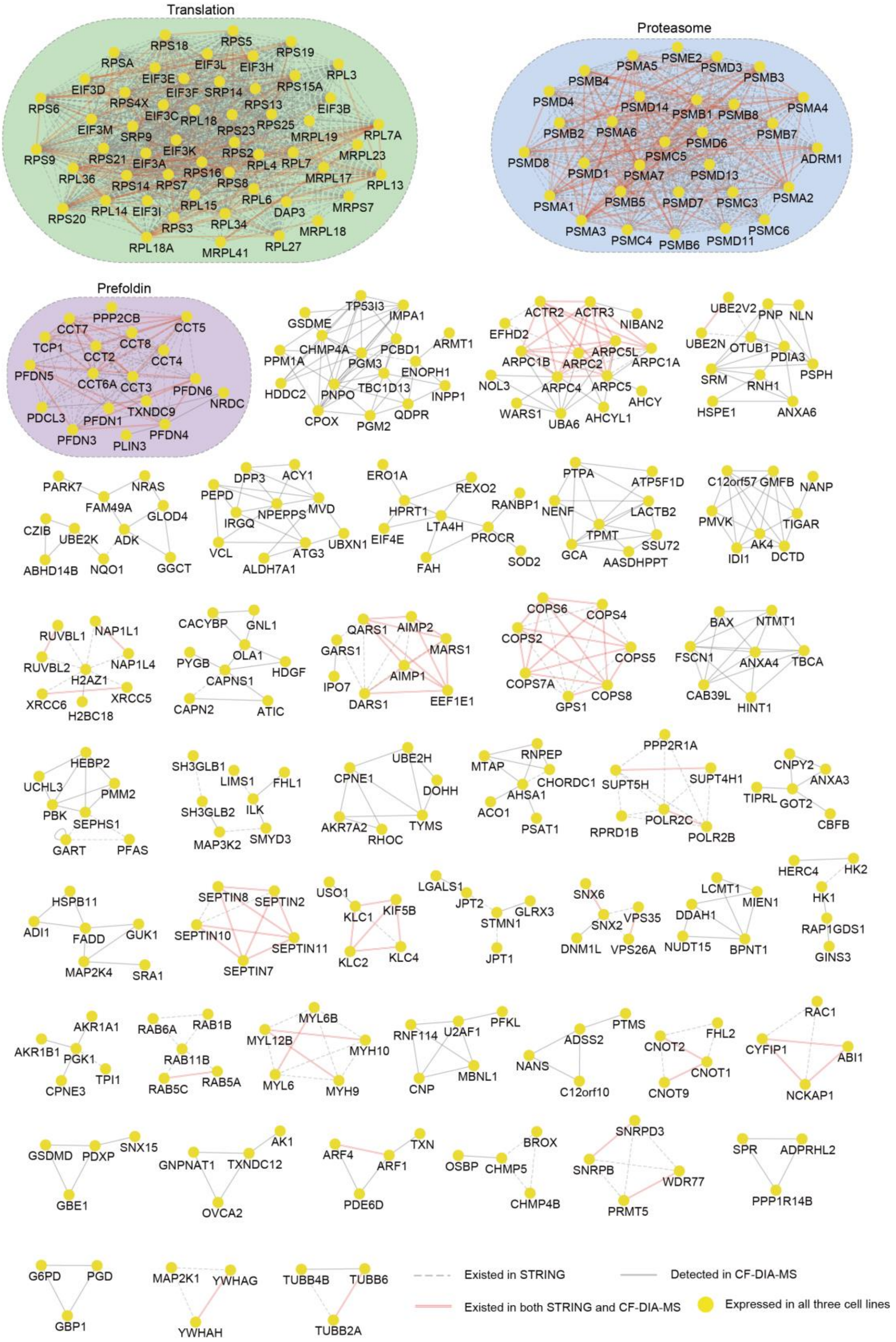

Translation
RPS18
RPS5
RPS19
RPSA
EIF3L
EIF3H
RPL3
EIF3D
EIF3E
EIF3F
SRP14
RPS15A
RPS6
RPS4X
RPS13
EIF3B
EIF3C
RPL18
RPS25
EIF3M
SRP9
RPS23
MRPL19
RPL7A
EIF3K
RPS2
MRPL23
RPS9
RPS21
EIF3A
RPL4
RPL7
RPL36
RPS16
MRPL17
RPS14
RPS7
RPS8
RPL13
RPL6
RPS20
RPL14
EIF3I
RPL15
DAP3
MRPS7
RPS3
RPL34
MRPL18
RPL18A
MRPL41
RPL27
Proteasome
PSME2
PSMA5
PSMD3
PSMB4
PSMB3
PSMD4
PSMD14
PSMA4
PSMB1
PSMB8
PSMB2
PSMA6
PSMD6
PSMB7
PSMC5
ADRM1
PSMD8
PSMD1
PSMA7
PSMD13
PSMB5
PSMD7
PSMC3
PSMA2
PSMA1
PSMA3
PSMC4
PSMB6
PSMD11
PSMC6
Prefoldin
PPP2CB
CCT7
CCT5
CCT8
TCP1
CCT2
CCT4
PFDN5
CCT6A
CCT3
PFDN6
NRDC
TXNDC9
PDCL3
PFDN1
PFDN4
PFDN3
PLIN3
TP53I3
GSDME
IMPA1
CHMP4A
PCBD1
ARMT1
PPM1A
PGM3
ENOPH1
HDDC2
PNPO
TBC1D13
INPP1
CPOX
QDPR
PGM2
ACTR2
ACTR3
EFHD2
NIBAN2
ARPC5L
ARPC1B
ARPC2
ARPC1A
NOL3
ARPC4
ARPC5
AHCY
WARS1
UBA6
AHCYL1
UBE2V2
PNP
NLN
UBE2N
OTUB1
PDIA3
SRM
RNH1
PSPH
HSPE1
ANXA6
PARK7
NRAS
FAM49A
GLOD4
CZIB
UBE2K
ADK
ABHD14B
NQO1
GGCT
DPP3
ACY1
PEPD
NPEPPS
MVD
IRGQ
UBXN1
VCL
ATG3
ALDH7A1
ERO1A
REXO2
RANBP1
HPRT1
LTA4H
EIF4E
PROCR
FAH
SOD2
PTPA
ATP5F1D
NENF
LACTB2
TPMT
SSU72
GCA
AASDHPPT
C12orf57
GMFB
NANP
PMVK
TIGAR
AK4
IDI1
DCTD
NAP1L1
RUVBL1
RUVBL2
NAP1L4
H2AZ1
XRCC6
XRCC5
H2BC18
CACYBP
GNL1
OLA1
PYGB
HDGF
CAPNS1
CAPN2
ATIC
QARS1
AIMP2
GARS1
MARS1
AIMP1
IPO7
DARS1
EEF1E1
COPS6
COPS4
COPS2
COPS5
COPS7A
COPS8
GPS1
BAX
NTMT1
FSCN1
ANXA4
TBCA
CAB39L
HINT1
HEBP2
UCHL3
PMM2
PBK
SEPHS1
GART
PFAS
SH3GLB1
LIMS1
FHL1
ILK
SH3GLB2
MAP3K2
SMYD3
UBE2H
CPNE1
DOHH
AKR7A2
TYMS
RHOC
RNPEP
MTAP
CHORDC1
AHSA1
ACO1
PSAT1
PPP2R1A
SUPT4H1
SUPT5H
POLR2C
RPRD1B
POLR2B
CNPY2
ANXA3
TIPRL
GOT2
CBFB
HSPB11
ADI1
FADD
GUK1
MAP2K4
SRA1
SEPTIN8
SEPTIN2
SEPTIN10
SEPTIN11
SEPTIN7
USO1
KIF5B
KLC1
KLC2
KLC4
LGALS1
JPT2
GLRX3
STMN1
JPT1
SNX6
VPS35
SNX2
DNM1L
VPS26A
LCMT1
MIEN1
DDAH1
NUDT15
BPNT1
HERC4
HK2
HK1
RAP1GDS1
GINS3
AKR1A1
AKR1B1
PGK1
TPI1
CPNE3
RAB6A
RAB1B
RAB11B
RAB5C
RAB5A
MYL6B
MYL12B
MYH10
MYL6
MYH9
RNF114
U2AF1
PFKL
CNP
MBNL1
PTMS
ADSS2
NANS
C12orf10
CNOT2
FHL2
CNOT1
CNOT9
RAC1
CYFIP1
ABI1
NCKAP1
GSDMD
PDXP
SNX15
GBE1
AK1
GNPNAT1
TXNDC12
OVCA2
ARF4
TXN
ARF1
PDE6D
OSBP
BROX
CHMP5
CHMP4B
SNRPD3
SNRPB
WDR77
PRMT5
SPR
ADPRHL2
PPP1R14B
G6PD
PGD
GBP1
MAP2K1
YWHAG
YWHAH
TUBB4B
TUBB6
TUBB2A
Existed in STRING
Detected in CF-DIA-MS
Existed in both STRING and CF-DIA-MS
Expressed in all three cell lines

Supplementary Fig. 4 Differentially expressed protein-protein interactions in thyroid cancer cell lines. (A) Differentially expressed protein pairs between FTC238 versus Nthy-ori 3-1. Significant pairs were identified with an adjusted p-value (adjP) <0.05 and a fold change >1.5 or <−1.5. Upregulated pairs are shown in orange, downregulated pairs in blue, and non-significant pairs in grey. The corresponding gene symbols of the top five statistically significant differential interactions (adjP) are displayed in the figure, formatted as "gene symbol1_gene symbol2" Statistical significance was assessed using a likelihood ratio test, and it is interpreted as two-sided. P values were adjusted for multiple comparisons using the Benjamini-Hochberg procedure. The test statistic follows a chi-squared distribution with 1 degree of freedom under the null hypothesis. **(B)** Differentially expressed protein pairs between TPC-1 versus Nthy-ori 3-1. Significance thresholds and color scheme are consistent with panel A. **(C)** Hierarchical clustering of differential protein-protein interactions (PPIs) from FTC238 versus Nthy-ori 3-1. Cutting the dendrogram at height = 50 yielded 6 and 8 intersecting points of differential proteins shared between the two cell lines, respectively. **(D)** Hierarchical clustering of differential protein-protein interactions (PPIs) from TPC-1 versus Nthy-ori 3-1. There are 7 and 9 intersecting points in TPC-1 and Nthy-ori 3-1 at height = 50.

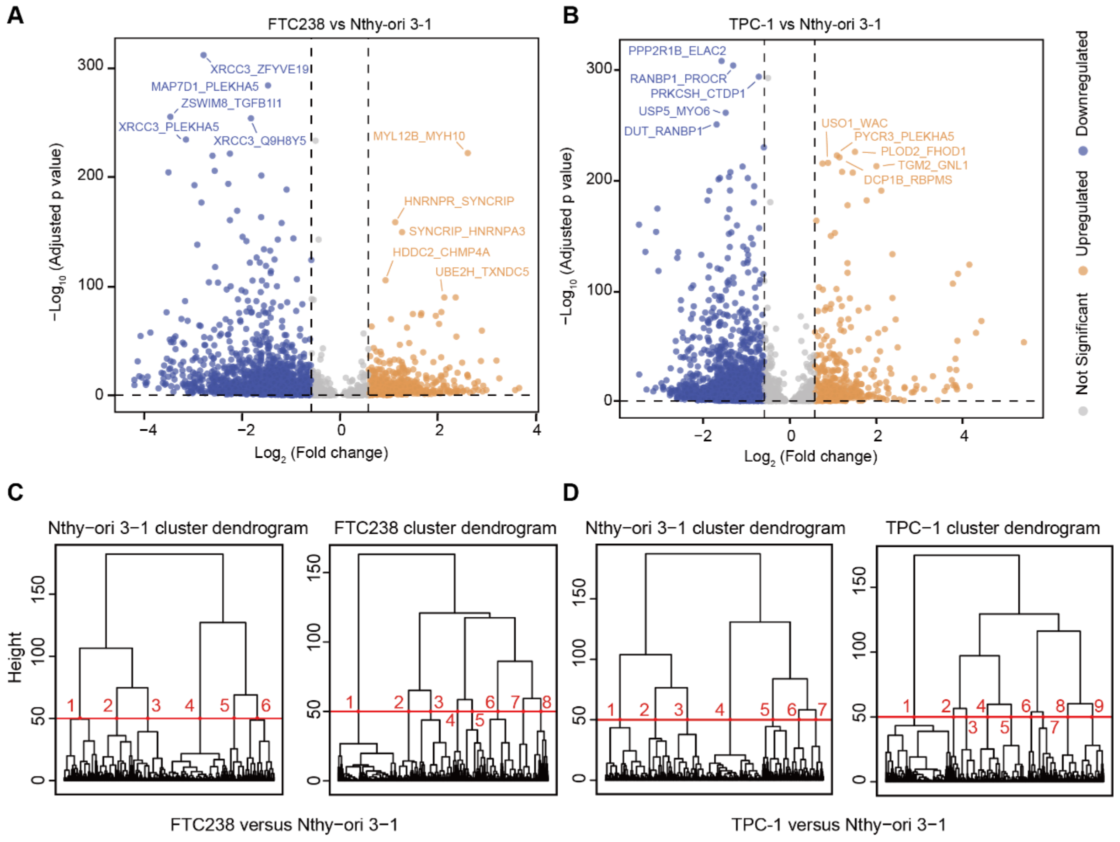

Supplementary Fig. 5 Differential protein interactions associated with the Prefoldin complex in TPC-1 versus Nthy-ori 3-1. (A) Prefoldin subunits form the clusters in the protein interaction network of Nthy-ori 3-1. The color of the node represents the averaged intensity across replicates within each cell line, with the color bar (from 0 to 0.1) indicating the intensity range across the displayed network. Grey edges for interactions identified by CF-DIA-MS, and red for those confirmed by both CF-DIA-MS and known complexes in the STRING database. **(B)** Prefoldin subunits form the clusters in the protein interaction network of TPC-1. The protein interactions show significantly differential expression between Nthy-ori 3-1. The scheme for nodes and edges remains consistent with that defined in panel A. **(C)** Heatmap of interacting proteins corresponding to the nodes displayed in Nthy-ori 3-1 (panel A). The color bar refers to normalized intensity. Protein and gene names are annotated above each column, with retention time displayed along the rows. **(D)** Heatmap of interacting proteins corresponding to the nodes displayed in TPC-1 (panel B). The visual scheme is the same as panel C.

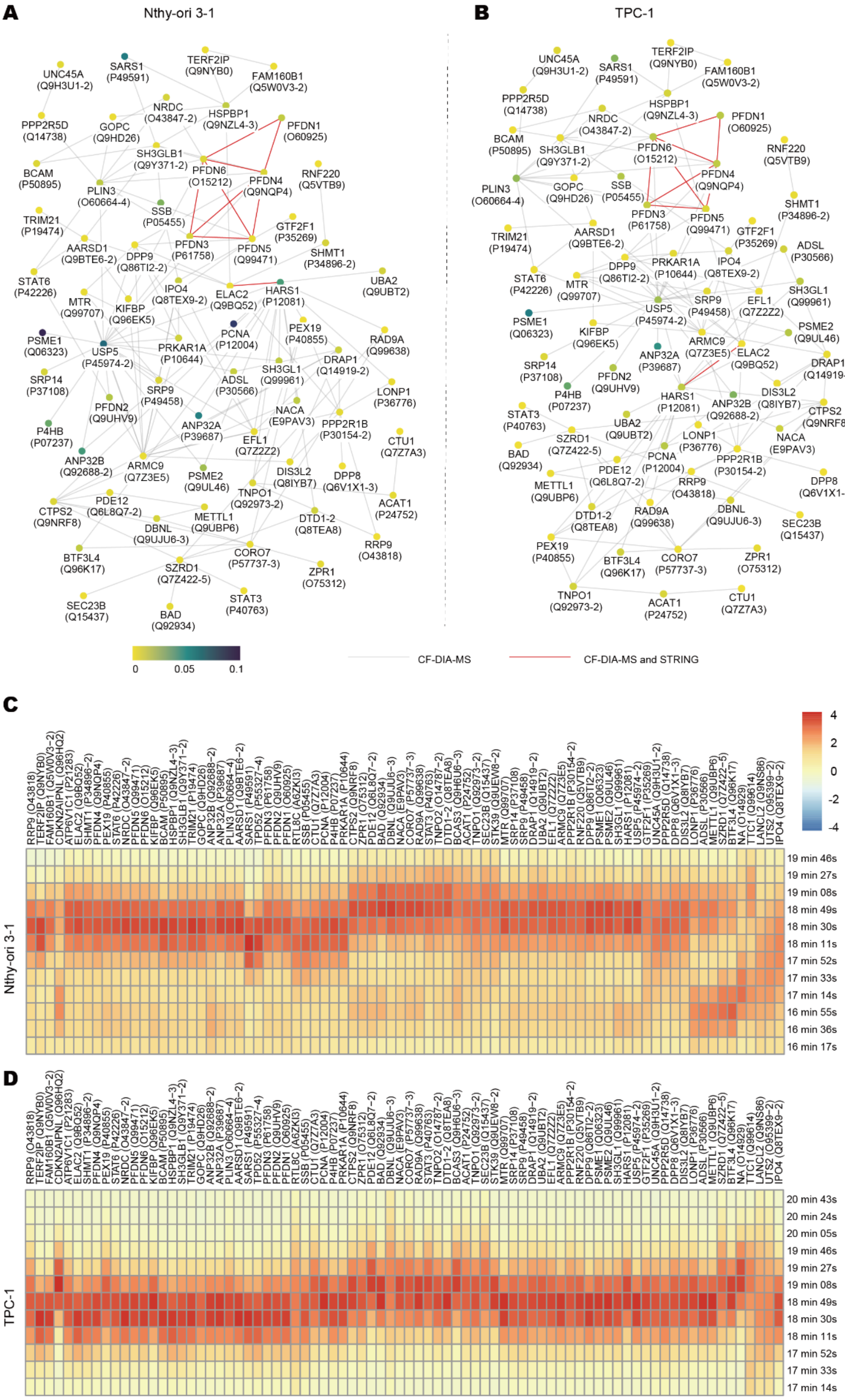
A
Nthy-ori 3-1
B
TPC-1
0
0.05
0.1
CF-DIA-MS
CF-DIA-MS and STRING
C
4
2
0
-2
-4
Nthy-ori 3-1
19 min 46s
19 min 27s
19 min 08s
18 min 49s
18 min 30s
18 min 11s
17 min 52s
17 min 33s
17 min 14s
16 min 55s
16 min 36s
16 min 17s
D
TPC-1
20 min 43s
20 min 24s
20 min 05s
19 min 46s
19 min 27s
19 min 08s
18 min 49s
18 min 30s
18 min 11s
17 min 52s
17 min 33s
17 min 14s

Supplementary Fig. 6 Differential protein interactions associated with the Prefoldin complex in FTC238 versus Nthy-ori 3-1. (A) Prefoldin subunits form the clusters in the protein interaction network of Nthy-ori 3-1. The scheme of node and edge is consistent with supplementary Fig.5A. **(B)** Prefoldin subunits form the clusters in the protein interaction network of FTC238. The protein interactions show significantly differential expression between Nthy-ori 3-1. **(C)** Heatmap of interacting proteins corresponding to the nodes displayed in Nthy-ori 3-1 (panel A). **(D)** Heatmap of interacting proteins corresponding to the nodes displayed in FTC238 (panel B).

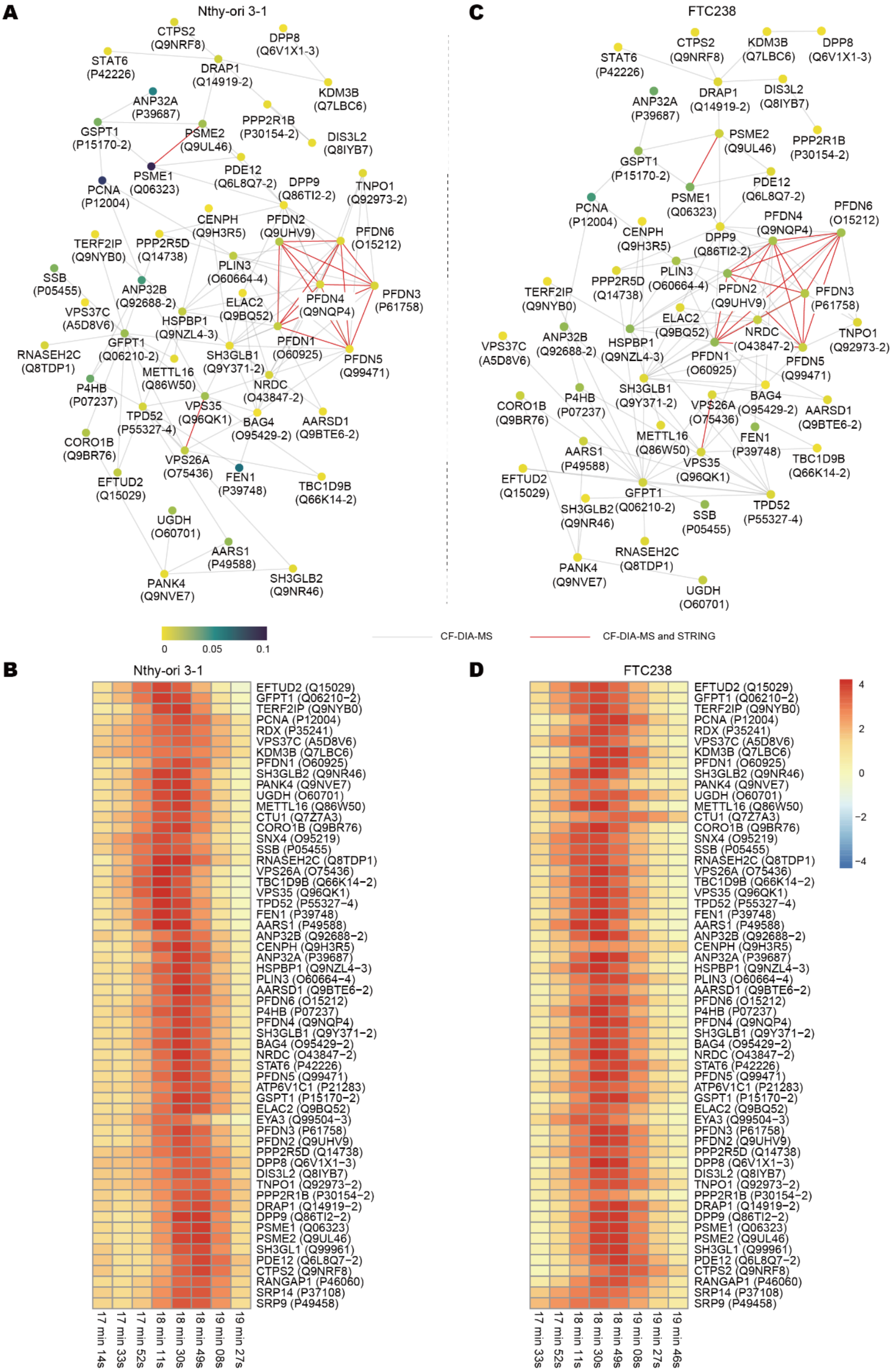
A
Nthy-ori 3-1
CTPS2 (Q9NRF8)
DPP8 (Q6V1X1-3)
STAT6 (P42226)
DRAP1 (Q14919-2)
KDM3B (Q7LBC6)
ANP32A (P39687)
PPP2R1B (P30154-2)
GSPT1 (P15170-2)
PSME2 (Q9UL46)
DIS3L2 (Q8IYB7)
PSME1 (Q06323)
PDE12 (Q6L8Q7-2)
PCNA (P12004)
DPP9 (Q86TI2-2)
TNPO1 (Q92973-2)
CENPH (Q9H3R5)
PFDN2 (Q9UHV9)
PFDN6 (O15212)
TERF2IP (Q9NYB0)
PPP2R5D (Q14738)
PLIN3 (O60664-4)
SSB (P05455)
ANP32B (Q92688-2)
PFDN4 (Q9NQP4)
PFDN3 (P61758)
VPS37C (A5D8V6)
ELAC2 (Q9BQ52)
HSPBP1 (Q9NZL4-3)
GFPT1 (Q06210-2)
PFDN1 (O60925)
RNASEH2C (Q8TDP1)
SH3GLB1 (Q9Y371-2)
PFDN5 (Q99471)
METTL16 (Q86W50)
P4HB (P07237)
NRDC (O43847-2)
VPS35 (Q96QK1)
TPD52 (P55327-4)
BAG4 (O95429-2)
AARSD1 (Q9BTE6-2)
CORO1B (Q9BR76)
VPS26A (O75436)
FEN1 (P39748)
EFTUD2 (Q15029)
TBC1D9B (Q66K14-2)
UGDH (O60701)
AARS1 (P49588)
PANK4 (Q9NVE7)
SH3GLB2 (Q9NR46)
C
FTC238
CTPS2 (Q9NRF8)
KDM3B (Q7LBC6)
DPP8 (Q6V1X1-3)
STAT6 (P42226)
DRAP1 (Q14919-2)
DIS3L2 (Q8IYB7)
ANP32A (P39687)
PSME2 (Q9UL46)
PPP2R1B (P30154-2)
GSPT1 (P15170-2)
PDE12 (Q6L8Q7-2)
PSME1 (Q06323)
PFDN6 (O15212)
PCNA (P12004)
PFDN4 (Q9NQP4)
CENPH (Q9H3R5)
DPP9 (Q86TI2-2)
PLIN3 (O60664-4)
TERF2IP (Q9NYB0)
PPP2R5D (Q14738)
PFDN2 (Q9UHV9)
PFDN3 (P61758)
ELAC2 (Q9BQ52)
VPS37C (A5D8V6)
ANP32B (Q92688-2)
HSPBP1 (Q9NZL4-3)
NRDC (O43847-2)
TNPO1 (Q92973-2)
PFDN1 (O60925)
PFDN5 (Q99471)
CORO1B (Q9BR76)
P4HB (P07237)
SH3GLB1 (Q9Y371-2)
VPS26A (O75436)
BAG4 (O95429-2)
AARSD1 (Q9BTE6-2)
METTL16 (Q86W50)
FEN1 (P39748)
AARS1 (P49588)
VPS35 (Q96QK1)
TBC1D9B (Q66K14-2)
EFTUD2 (Q15029)
GFPT1 (Q06210-2)
SH3GLB2 (Q9NR46)
TPD52 (P55327-4)
SSB (P05455)
RNASEH2C (Q8TDP1)
PANK4 (Q9NVE7)
UGDH (O60701)
0
0.05
0.1
CF-DIA-MS
CF-DIA-MS and STRING
B
Nthy-ori 3-1
D
FTC238
EFTUD2 (Q15029)
GFPT1 (Q06210−2)
TERF2IP (Q9NYB0)
PCNA (P12004)
RDX (P35241)
VPS37C (A5D8V6)
KDM3B (Q7LBC6)
PFDN1 (O60925)
SH3GLB2 (Q9NR46)
PANK4 (Q9NVE7)
UGDH (O60701)
METTL16 (Q86W50)
CTU1 (Q7Z7A3)
CORO1B (Q9BR76)
SNX4 (O95219)
SSB (P05455)
RNASEH2C (Q8TDP1)
VPS26A (O75436)
TBC1D9B (Q66K14−2)
VPS35 (Q96QK1)
TPD52 (P55327−4)
FEN1 (P39748)
AARS1 (P49588)
ANP32B (Q92688−2)
CENPH (Q9H3R5)
ANP32A (P39687)
HSPBP1 (Q9NZL4−3)
PLIN3 (O60664−4)
AARSD1 (Q9BTE6−2)
PFDN6 (O15212)
P4HB (P07237)
PFDN4 (Q9NQP4)
SH3GLB1 (Q9Y371−2)
BAG4 (O95429−2)
NRDC (O43847−2)
STAT6 (P42226)
PFDN5 (Q99471)
ATP6V1C1 (P21283)
GSPT1 (P15170−2)
ELAC2 (Q9BQ52)
EYA3 (Q99504−3)
PFDN3 (P61758)
PFDN2 (Q9UHV9)
PPP2R5D (Q14738)
DPP8 (Q6V1X1−3)
DIS3L2 (Q8IYB7)
TNPO1 (Q92973−2)
PPP2R1B (P30154−2)
DRAP1 (Q14919−2)
DPP9 (Q86TI2−2)
PSME1 (Q06323)
PSME2 (Q9UL46)
SH3GL1 (Q99961)
PDE12 (Q6L8Q7−2)
CTPS2 (Q9NRF8)
RANGAP1 (P46060)
SRP14 (P37108)
SRP9 (P49458)
17 min 14s
17 min 33s
17 min 52s
18 min 11s
18 min 30s
18 min 49s
19 min 08s
19 min 27s
17 min 33s
17 min 52s
18 min 11s
18 min 30s
18 min 49s
19 min 08s
19 min 27s
19 min 46s
4
2
0
−2
−4

Supplementary Fig. 7 Differential protein interactions associated with the proteasome in FTC238 versus Nthy-ori 3-1. (A) Proteasome related proteins form the clusters in the protein interaction network of Nthy-ori 3-1. The scheme of node and edge is consistent with supplementary Fig.5A. **(B)** Proteasome related proteins form clusters in the protein interaction network of FTC238. The protein interactions show significantly differential expression between Nthy-ori 3-1. **(C)** Heatmap of interacting proteins corresponding to the nodes displayed in Nthy-ori 3-1 (panel A). **(D)** Heatmap of interacting proteins corresponding to the nodes displayed in FTC238 (panel B).

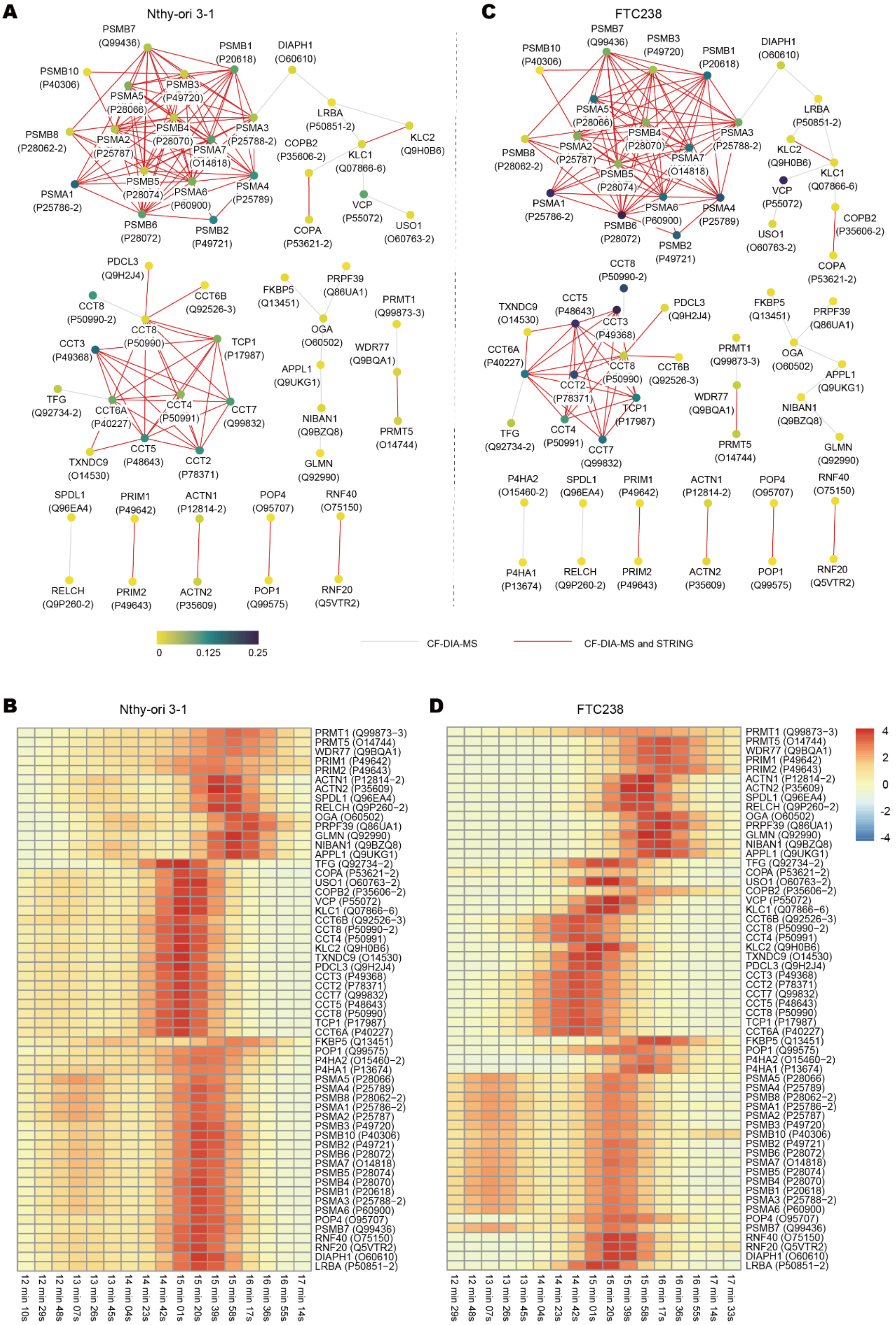
A
Nthy-ori 3-1
PSMB7 (Q99436)
PSMB1 (P20618)
DIAPH1 (O60610)
PSMB10 (P40306)
PSMB3 (P49720)
PSMA5 (P28066)
LRBA (P50851-2)
PSMB4 (P28070)
PSMA3 (P25788-2)
KLC2 (Q9H0B6)
PSMB8 (P28062-2)
PSMA2 (P25787)
PSMA7 (O14818)
COPB2 (P35606-2)
KLC1 (Q07866-6)
PSMB5 (P28074)
PSMA6 (P60900)
PSMA4 (P25789)
PSMA1 (P25786-2)
VCP (P55072)
USO1 (O60763-2)
PSMB6 (P28072)
PSMB2 (P49721)
COPA (P53621-2)
PDCL3 (Q9H2J4)
CCT6B (Q92526-3)
FKBP5 (Q13451)
PRPF39 (Q86UA1)
CCT8 (P50990-2)
PRMT1 (Q99873-3)
CCT8 (P50990)
OGA (O60502)
CCT3 (P49368)
TCP1 (P17987)
WDR77 (Q9BQA1)
APPL1 (Q9UKG1)
TFG (Q92734-2)
CCT6A (P40227)
CCT4 (P50991)
CCT7 (Q99832)
NIBAN1 (Q9BZQ8)
PRMT5 (O14744)
TXNDC9 (O14530)
CCT5 (P48643)
CCT2 (P78371)
GLMN (Q92990)
SPDL1 (Q96EA4)
PRIM1 (P49642)
ACTN1 (P12814-2)
POP4 (O95707)
RNF40 (O75150)
RELCH (Q9P260-2)
PRIM2 (P49643)
ACTN2 (P35609)
POP1 (Q99575)
RNF20 (Q5VTR2)
C
FTC238
PSMB7 (Q99436)
PSMB3 (P49720)
PSMB1 (P20618)
DIAPH1 (O60610)
PSMB10 (P40306)
PSMA5 (P28066)
LRBA (P50851-2)
PSMB4 (P28070)
PSMA3 (P25788-2)
PSMB8 (P28062-2)
PSMA2 (P25787)
PSMA7 (O14818)
KLC2 (Q9H0B6)
PSMB5 (P28074)
KLC1 (Q07866-6)
VCP (P55072)
PSMA1 (P25786-2)
PSMA6 (P60900)
PSMA4 (P25789)
COPB2 (P35606-2)
PSMB6 (P28072)
USO1 (O60763-2)
PSMB2 (P49721)
COPA (P53621-2)
CCT8 (P50990-2)
TXNDC9 (O14530)
CCT5 (P48643)
PDCL3 (Q9H2J4)
FKBP5 (Q13451)
PRPF39 (Q86UA1)
CCT3 (P49368)
CCT6A (P40227)
CCT8 (P50990)
CCT6B (Q92526-3)
PRMT1 (Q99873-3)
OGA (O60502)
APPL1 (Q9UKG1)
CCT2 (P78371)
WDR77 (Q9BQA1)
NIBAN1 (Q9BZQ8)
TCP1 (P17987)
TFG (Q92734-2)
CCT4 (P50991)
CCT7 (Q99832)
PRMT5 (O14744)
GLMN (Q92990)
P4HA2 (O15460-2)
SPDL1 (Q96EA4)
PRIM1 (P49642)
ACTN1 (P12814-2)
POP4 (O95707)
RNF40 (O75150)
P4HA1 (P13674)
RELCH (Q9P260-2)
PRIM2 (P49643)
ACTN2 (P35609)
POP1 (Q99575)
RNF20 (Q5VTR2)
0
0.125
0.25
CF-DIA-MS
CF-DIA-MS and STRING
B
Nthy-ori 3-1
D
FTC238
PRMT1 (Q99873-3)
PRMT5 (O14744)
WDR77 (Q9BQA1)
PRIM1 (P49642)
PRIM2 (P49643)
ACTN1 (P12814-2)
ACTN2 (P35609)
SPDL1 (Q96EA4)
RELCH (Q9P260-2)
OGA (O60502)
PRPF39 (Q86UA1)
GLMN (Q92990)
NIBAN1 (Q9BZQ8)
APPL1 (Q9UKG1)
TFG (Q92734-2)
COPA (P53621-2)
USO1 (O60763-2)
COPB2 (P35606-2)
VCP (P55072)
KLC1 (Q07866-6)
CCT6B (Q92526-3)
CCT8 (P50990-2)
CCT4 (P50991)
KLC2 (Q9H0B6)
TXNDC9 (O14530)
PDCL3 (Q9H2J4)
CCT3 (P49368)
CCT2 (P78371)
CCT7 (Q99832)
CCT5 (P48643)
CCT8 (P50990)
TCP1 (P17987)
CCT6A (P40227)
FKBP5 (Q13451)
POP1 (Q99575)
P4HA2 (O15460-2)
P4HA1 (P13674)
PSMA5 (P28066)
PSMA4 (P25789)
PSMB8 (P28062-2)
PSMA1 (P25786-2)
PSMA2 (P25787)
PSMB3 (P49720)
PSMB10 (P40306)
PSMB2 (P49721)
PSMB6 (P28072)
PSMA7 (O14818)
PSMB5 (P28074)
PSMB4 (P28070)
PSMB1 (P20618)
PSMA3 (P25788-2)
PSMA6 (P60900)
POP4 (O95707)
PSMB7 (Q99436)
RNF40 (O75150)
RNF20 (Q5VTR2)
DIAPH1 (O60610)
LRBA (P50851-2)
12 min 10s
12 min 29s
12 min 48s
13 min 07s
13 min 26s
13 min 45s
14 min 04s
14 min 23s
14 min 42s
15 min 01s
15 min 20s
15 min 39s
15 min 58s
16 min 17s
16 min 36s
16 min 55s
17 min 14s
17 min 33s
4
2
0
-2
-4

Supplementary Fig. 8 Differential protein interactions associated with proteostasis and cytoskeletal complexes in FTC238 versus Nthy-ori 3-1. (A) Proteostasis and cytoskeletal complexes related proteins form the protein interaction networks (PINs) in Nthy-ori 3-1. Proteasome complex, translation elongation factor complex and myosin/actomyosin complex in the PINs. The scheme of node and edge is consistent with supplementary Fig.5A. **(B)** Proteostasis and cytoskeletal complexes related proteins form the protein interaction networks (PINs) in FTC238.The protein interactions show significantly differential expression between Nthy-ori 3-1. **(C)** Heatmap of interacting proteins corresponding to the nodes displayed in Nthy-ori 3-1 (panel A). **(D)** Heatmap of interacting proteins corresponding to the nodes displayed in FTC238 (panel B).

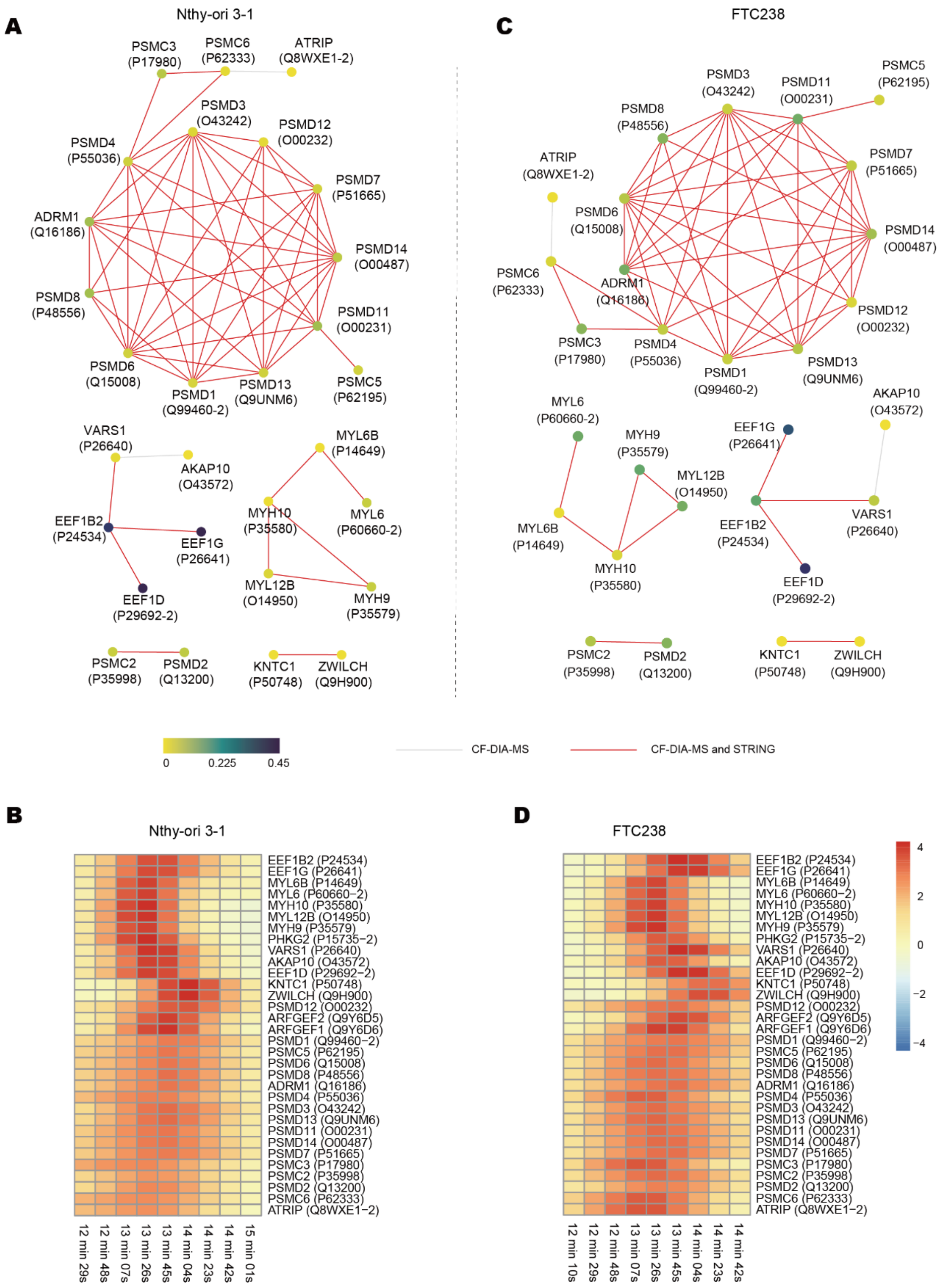
A
Nthy-ori 3-1
PSMC3 (P17980)
PSMC6 (P62333)
ATRIP (Q8WXE1-2)
PSMD3 (O43242)
PSMD12 (O00232)
PSMD4 (P55036)
PSMD7 (P51665)
ADRM1 (Q16186)
PSMD14 (O00487)
PSMD8 (P48556)
PSMD11 (O00231)
PSMD6 (Q15008)
PSMC5 (P62195)
PSMD1 (Q99460-2)
PSMD13 (Q9UNM6)
VARS1 (P26640)
AKAP10 (O43572)
MYL6B (P14649)
EEF1B2 (P24534)
EEF1G (P26641)
MYH10 (P35580)
MYL6 (P60660-2)
EEF1D (P29692-2)
MYL12B (O14950)
MYH9 (P35579)
PSMC2 (P35998)
PSMD2 (Q13200)
KNTC1 (P50748)
ZWILCH (Q9H900)
C
FTC238
PSMC5 (P62195)
PSMD3 (O43242)
PSMD11 (O00231)
PSMD8 (P48556)
PSMD7 (P51665)
ATRIP (Q8WXE1-2)
PSMD6 (Q15008)
PSMD14 (O00487)
PSMC6 (P62333)
ADRM1 (Q16186)
PSMD12 (O00232)
PSMC3 (P17980)
PSMD4 (P55036)
PSMD1 (Q99460-2)
PSMD13 (Q9UNM6)
AKAP10 (O43572)
MYL6 (P60660-2)
MYH9 (P35579)
EEF1G (P26641)
MYL12B (O14950)
MYL6B (P14649)
EEF1B2 (P24534)
VARS1 (P26640)
MYH10 (P35580)
EEF1D (P29692-2)
PSMC2 (P35998)
PSMD2 (Q13200)
KNTC1 (P50748)
ZWILCH (Q9H900)
0
0.225
0.45
CF-DIA-MS
CF-DIA-MS and STRING
B
Nthy-ori 3-1
EEF1B2 (P24534)
EEF1G (P26641)
MYL6B (P14649)
MYL6 (P60660−2)
MYH10 (P35580)
MYL12B (O14950)
MYH9 (P35579)
PHKG2 (P15735−2)
VARS1 (P26640)
AKAP10 (O43572)
EEF1D (P29692−2)
KNTC1 (P50748)
ZWILCH (Q9H900)
PSMD12 (O00232)
ARFGEF2 (Q9Y6D5)
ARFGEF1 (Q9Y6D6)
PSMD1 (Q99460−2)
PSMC5 (P62195)
PSMD6 (Q15008)
PSMD8 (P48556)
ADRM1 (Q16186)
PSMD4 (P55036)
PSMD3 (O43242)
PSMD13 (Q9UNM6)
PSMD11 (O00231)
PSMD14 (O00487)
PSMD7 (P51665)
PSMC3 (P17980)
PSMC2 (P35998)
PSMD2 (Q13200)
PSMC6 (P62333)
ATRIP (Q8WXE1−2)
12 min 29s
12 min 48s
13 min 07s
13 min 26s
13 min 45s
14 min 04s
14 min 23s
14 min 42s
15 min 01s
D
FTC238
EEF1B2 (P24534)
EEF1G (P26641)
MYL6B (P14649)
MYL6 (P60660−2)
MYH10 (P35580)
MYL12B (O14950)
MYH9 (P35579)
PHKG2 (P15735−2)
VARS1 (P26640)
AKAP10 (O43572)
EEF1D (P29692−2)
KNTC1 (P50748)
ZWILCH (Q9H900)
PSMD12 (O00232)
ARFGEF2 (Q9Y6D5)
ARFGEF1 (Q9Y6D6)
PSMD1 (Q99460−2)
PSMC5 (P62195)
PSMD6 (Q15008)
PSMD8 (P48556)
ADRM1 (Q16186)
PSMD4 (P55036)
PSMD3 (O43242)
PSMD13 (Q9UNM6)
PSMD11 (O00231)
PSMD14 (O00487)
PSMD7 (P51665)
PSMC3 (P17980)
PSMC2 (P35998)
PSMD2 (Q13200)
PSMC6 (P62333)
ATRIP (Q8WXE1−2)
12 min 10s
12 min 29s
12 min 48s
13 min 07s
13 min 26s
13 min 45s
14 min 04s
14 min 23s
14 min 42s
4
2
0
−2
−4

Supplementary Fig. 9 Predicted protein interactions in TPC-1 and Nthy-ori 3-1. (A) Predicted interactions (HK1 and TGM2) form the clusters in the protein interaction network of Nthy-ori 3-1. The scheme of node and edge is the same as supplementary Fig.5A, while the color bar is from 0 to 0.09. **(B)** Predicted interactions (HK1 and TGM2) form the clusters in the protein interaction network of TPC-1.

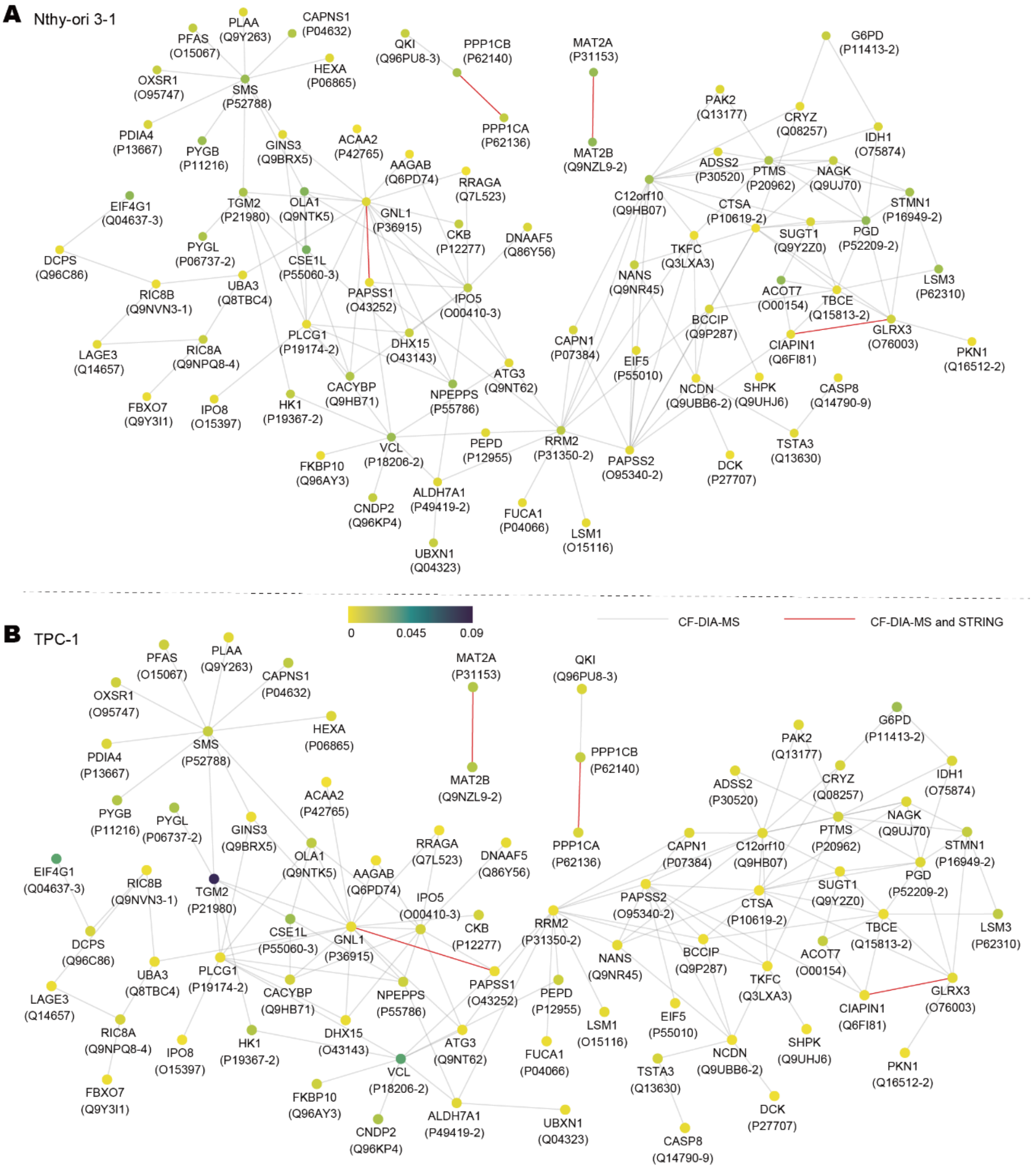

Supplementary Fig. 10 Predicted protein interactions in TPC-1 and Nthy-ori 3-1. (A) Predicted interactions (HK1 and TGM2) form the clusters in the protein interaction network of Nthy-ori 3-1. Heatmap of the interacting proteins corresponding to the nodes displayed in Nthy-ori 3-1 (supplementary Fig. 9A). **(B)** Heatmap of interacting proteins corresponding to the nodes displayed in FTC238 (supplementary Fig. 9B).

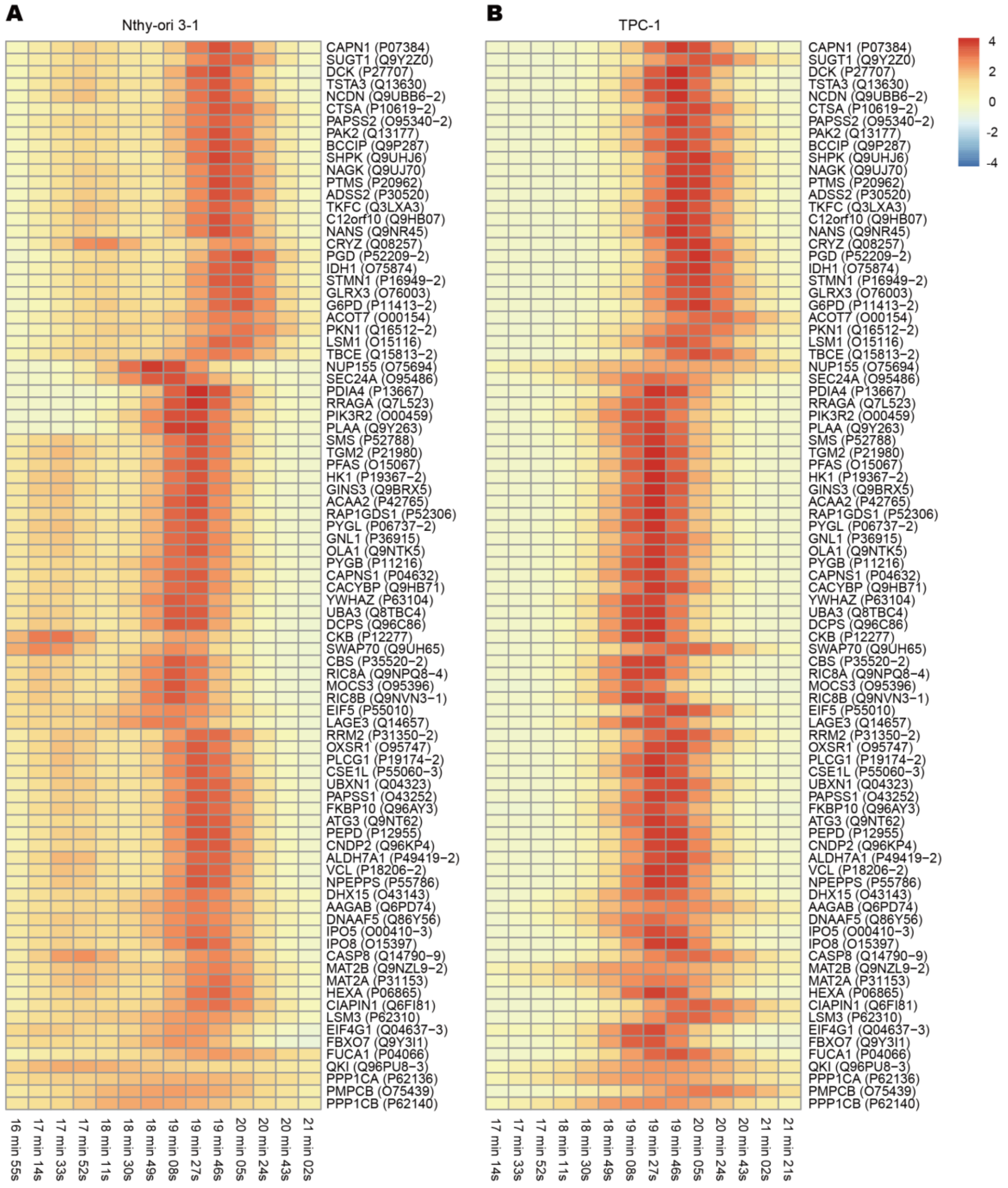

Supplement: Supplementary file 1 — Supplementary Information [file 41467_2026_68686_MOESM1_ESM.pdf]
