# Supplementary material for: ProteoAutoNet: high-throughput co-eluted protein analysis with robotics and machine learning
Source: Nat Commun. 2026 Jan 22;17:1949. doi: 10.1038/s41467-026-68686-9 (PMC12929803; doi:10.1038/s41467-026-68686-9)
Supplement: Supplementary file 6 — Reporting Summary [file 41467_2026_68686_MOESM6_ESM.pdf]

nature portfolio

Corresponding author(s): Rui Sun, Yi Chen, Tiannan Guo

Last updated by author(s): December 17, 2025 (by Mengge LYU)

Reporting Summary

Nature Portfolio wishes to improve the reproducibility of the work that we publish. This form provides structure for consistency and transparency in reporting. For further information on Nature Portfolio policies, see our Editorial Policies and the Editorial Policy Checklist.

Statistics

For all statistical analyses, confirm that the following items are present in the figure legend, table legend, main text, or Methods section.

n/a	Confirmed	
☐	☒	The exact sample size (*n*) for each experimental group/condition, given as a discrete number and unit of measurement
☐	☒	A statement on whether measurements were taken from distinct samples or whether the same sample was measured repeatedly
☐	☒	The statistical test(s) used AND whether they are one- or two-sided<br>*Only common tests should be described solely by name; describe more complex techniques in the Methods section.*
☒	☐	A description of all covariates tested
☐	☒	A description of any assumptions or corrections, such as tests of normality and adjustment for multiple comparisons
☐	☒	A full description of the statistical parameters including central tendency (e.g. means) or other basic estimates (e.g. regression coefficient) AND variation (e.g. standard deviation) or associated estimates of uncertainty (e.g. confidence intervals)
☐	☒	For null hypothesis testing, the test statistic (e.g. *F*, *t*, *r*) with confidence intervals, effect sizes, degrees of freedom and *P* value noted<br>*Give P values as exact values whenever suitable.*
☒	☐	For Bayesian analysis, information on the choice of priors and Markov chain Monte Carlo settings
☐	☒	For hierarchical and complex designs, identification of the appropriate level for tests and full reporting of outcomes
☐	☒	Estimates of effect sizes (e.g. Cohen's *d*, Pearson's *r*), indicating how they were calculated

Our web collection on statistics for biologists contains articles on many of the points above.

Software and code

Policy information about availability of computer code

Data collection	Peptides from fractional samples were separated using an Ultimate 3000 nanoLC system (Thermo Fisher Scientific) at 300 nL/min with an effective 30-min gradient. Mobile phase A consisted of 0.1% (v/v) formic acid in water, and mobile phase B consisted of 0.1% (v/v) formic acid in 80% acetonitrile. The column was equilibrated at 7% buffer B, and peptides were eluted with a 30-min effective gradient increasing from 7% to 28% buffer B over 30 min (from 4 to 34 min). Eluted peptides were ionized and analyzed using a Q-Exactive HF mass spectrometer (Thermo Fisher Scientific) operated in data-independent acquisition (DIA) mode. A full MS scan was acquired over m/z 390–1010 at a resolution of 60,000, with an automatic gain control (AGC) target of 3e6 and a maximum injection time of 80 ms. After the full MS scan, 24 MS/MS scans were acquired. Each MS/MS scan was acquired at a resolution of 30,000, with an AGC target of 1e6 and a maximum injection time set to auto.<br>We acquired a total of 576 analyzable DIA files, including 540 co-fractionated samples from three cell lines with three SEC replicates (60 SEC fractions per replicate collected every 19 seconds between 9-28 minutes), 27 unfractionated quality control samples of automated platform (three technical replicates per SEC replicate), and 9 batch control injections of pooled three cell line mixtures for MS acquisition.The details were displayed in Methods. The proteomics data, and sample information for all proteins identified are deposited in PRIDE under the accession number PXD059608 (https://www.ebi.ac.uk/pride/archive/projects/PXD059608).
Data analysis	DIA raw files were analyzed using DIA-NN 1.8 (default settings). The data analysis in this study was performed using R 4.3.0 and Python 3.9.21. Network visualization was conducted with Cytoscape software (v3.10.3, based on Java 17.0.5). The details were displayed in Materials and Methods. The computational framework ProteoAutoNet, including R and Python scripts along with visualization files, is publicly available at: https://github.com/lyumengge/ProteoAutoNet. The code and example files that were used in this study are publicly available on Zenodo at https://doi.org/10.5281/zenodo.17931374.

For manuscripts utilizing custom algorithms or software that are central to the research but not yet described in published literature, software must be made available to editors and reviewers. We strongly encourage code deposition in a community repository (e.g. GitHub). See the Nature Portfolio guidelines for submitting code & software for further information.

Data

Policy information about availability of data

All manuscripts must include a data availability statement. This statement should provide the following information, where applicable:

- Accession codes, unique identifiers, or web links for publicly available datasets
- A description of any restrictions on data availability
- For clinical datasets or third party data, please ensure that the statement adheres to our policy

The proteomics data, the spectral library, and information for all proteins identified are deposited in PRIDE under the accession number PXD059608. The computational framework ProteoAutoNet, including R and Python scripts and visualized files, is publicly available at: https://github.com/lyumengge/ProteoAutoNet

Research involving human participants, their data, or biological material

Policy information about studies with human participants or human data. See also policy information about sex, gender (identity/presentation), and sexual orientation and race, ethnicity and racism.

Reporting on sex and gender	not applicable
Reporting on race, ethnicity, or other socially relevant groupings	not applicable
Population characteristics	not applicable
Recruitment	not applicable
Ethics oversight	not applicable

Note that full information on the approval of the study protocol must also be provided in the manuscript.

Field-specific reporting

Please select the one below that is the best fit for your research. If you are not sure, read the appropriate sections before making your selection.

☒ Life sciences ☐ Behavioural & social sciences ☐ Ecological, evolutionary & environmental sciences

For a reference copy of the document with all sections, see nature.com/documents/nr-reporting-summary-flat.pdf

Life sciences study design

All studies must disclose on these points even when the disclosure is negative.

Sample size	The study utilized three distinct human thyroid-derived cell lines: one normal epithelial cell line (Nthy-ori 3-1) and two carcinoma cell lines (TPC-1 and FTC-238). For quality control during the large-scale run, nine unfractionated mixtures from the three cell lines were processed and analyzed alongside the 540 fractionated samples. The sample size for the main co-fractionation experiment consisted of three size exclusion chromatography (SEC) replicates per cell line, generating 60 fractions each (totaling 567 fractions).
Data exclusions	No data were explicitly excluded from the analyses described in the provided methods.
Replication	Each cell line had three biological replicates. Besides, for quality control, three technical replicates of the unfractionated quality control samples were analyzed per SEC replicate. Blank and standard protein runs were repeated every three sample injections to ensure consistent instrument performance.
Randomization	The provided methodology does not describe a specific randomization procedure for the processing of samples or the order of MS analysis.
Blinding	The provided methodology does not mention any blinding protocols.

Reporting for specific materials, systems and methods

We require information from authors about some types of materials, experimental systems and methods used in many studies. Here, indicate whether each material, system or method listed is relevant to your study. If you are not sure if a list item applies to your research, read the appropriate section before selecting a response.

Materials & experimental systems

n/a	Involved in the study
☒	☐ Antibodies
☐	☒ Eukaryotic cell lines
☒	☐ Palaeontology and archaeology
☒	☐ Animals and other organisms
☒	☐ Clinical data
☒	☐ Dual use research of concern
☒	☐ Plants

Methods

n/a	Involved in the study
☒	☐ ChIP-seq
☒	☐ Flow cytometry
☒	☐ MRI-based neuroimaging

Eukaryotic cell lines

Policy information about cell lines and Sex and Gender in Research

Cell line source(s)	All cell lines used in this study are established human thyroid cancer or normal thyroid cell lines obtained from commercial sources:<br>Nthy-ori 3-1 (normal follicular epithelial cells), originally obtained from ECACC (catalog no. 90011609), purchased from Cellverse Co., Ltd. (iCell-h335);<br>TPC-1 (papillary thyroid carcinoma), originally deposited in DSMZ (catalog no. ACC 517), purchased from BeNa Culture Collection (BNCC337912);<br>FTC238 (follicular thyroid carcinoma with lung metastasis), originally from JCRB Cell Bank (catalog no. JCRB1542), purchased from MeisenCTCC (CTCC-007-0085).
Authentication	The cell lines were authenticated by short tandem repeat (STR) profiling at low passage numbers prior to the initiation of the experiments.
Mycoplasma contamination	The cell lines used in this study were confirmed to be free of mycoplasma contamination.
Commonly misidentified lines<br>(See ICLAC register)	None of the cell lines used in this study (Nthy-ori 3-1, TPC-1, FTC-238) are listed on the International Cell Line Authentication Committee (ICLAC) register of commonly misidentified cell lines.

Plants

Seed stocks	Not applicable
Novel plant genotypes	Not applicable
Authentication	Not applicable
